# Decreased Expression of Sam68 Is Associated with Insulin Resistance in Granulosa Cells from PCOS Patients

**DOI:** 10.3390/cells11182821

**Published:** 2022-09-09

**Authors:** Teresa Vilariño-García, Pilar Guadix, Mónica Dorado-Silva, Pascual Sánchez-Martín, Antonio Pérez-Pérez, Víctor Sánchez-Margalet

**Affiliations:** 1 Department of Medical Biochemistry and Molecular Biology and Immunology, Virgen Macarena University Hospital, School of Medicine, University of Seville, 41009 Seville, Spain; 2 Obstetrics and Gynecology Department, Virgen Macarena University Hospital, School of Medicine, University of Seville, 41009 Seville, Spain; 3 GINEMED, Assisted Reproduction Centre, 41010 Seville, Spain

**Keywords:** Sam68, insulin, polycystic ovary syndrome (PCOS), signaling pathways

## Abstract

Background and objective: Polycystic ovary syndrome (PCOS) is a complex metabolic disorder associated with ovulatory dysfunction, hyperandrogenism, obesity, and insulin resistance, which leads to subfertility. PCOS is the most frequent metabolic disorder in women and the major cause of infertility. Susceptibility to developing PCOS is determined by a complex interaction between environmental and genetic factors. Although different mechanisms have been proposed to explain PCOS manifestations, defects in insulin actions or in the insulin signaling pathways are central in the pathogenesis of the syndrome. However, the mechanisms (molecular players and signaling pathways) underlying its primary origin still remain an unsolved issue. Current research is increasingly focusing on the discovery of novel biomarkers to further elucidate the complex pathophysiology of PCOS. Sam68, an RNA-binding protein, is recruited to insulin signaling, mediating different insulin actions. We aimed to investigate the role of Sam68 in insulin signaling and the possible implications of Sam68 in the insulin resistance in PCOS. Materials and methods: Granulosa cells were taken from women with PCOS (n = 25) and healthy donors (n = 25) and, within the age range of 20 to 42 years, from GINEMED, Assisted Reproduction Centre, Seville, Spain. The Sam68 expression level was analyzed both by qPCR and immunoblot. Statistical significance was assessed by one-way ANOVA, followed by a post-hoc test. A *p* value of < 0.05 was considered statistically significant. Results: We found that insulin stimulation increases the phosphorylation and expression level of Sam68 in granulosa cells from normal donors. The downregulation of Sam68 expression resulted in a lower activation of both the MAPK and the PI3K pathways in response to insulin. Moreover, the granulosa cells from the women with PCOS presented a lower expression of Sam68, as well as insulin receptor and insulin receptor substrate-1 (IRS-1). In these cells, the overexpression of Sam68 resulted in an increased activation of both the MAPK and the PI3K pathways in response to insulin. Conclusions: These results suggest the participation of Sam68 in insulin receptor signaling, mediating the insulin effect in granulosa cells, and they suggest the possible role of Sam68 in the insulin resistance of PCOS.

## 1. Introduction

Polycystic ovary syndrome (PCOS) is considered to be the most common endocrinopathy in women of reproductive age. Adverse reproductive outcomes include anovulation and infertility. The prevalence of PCOS is estimated to be between 6% and 25% [1,2], depending on the population analyzed. Women with PCOS are at higher risk of obesity and vice versa [3]. Moreover, insulin resistance and compensatory hyperinsulinaemia are considered to be highly prevalent among women with PCOS. Women with PCOS are also characterized by hyperandrogenaemia, hypothalamic pituitary–ovarian axis dysfunction, and deranged adipokines secretion from the adipose tissue, such as leptin.

The diagnosis of PCOS is based on a set of criteria defined by major expert conferences. In this sense, the Rotterdam criteria require the presence of two of the three criteria in question: oligo-/anovulation, clinical and/or biochemical hyperandrogenism, and an ultrasound image of the polycystic ovary [1]. This led to the recognition of PCOS as a syndrome with a variety of complex clinical phenotypes. In this context, a thorough understanding of PCOS pathophysiology and its association with reproductive and metabolic disturbances is essential for addressing women’s health and for expanding knowledge on how to treat this highly multifaceted syndrome [1].

Therefore, current research is increasingly focusing on the discovery of novel biomarkers to further elucidate the complex pathophysiology of PCOS. It has been reported that PCOS is a highly hereditary condition, with an approximately 70% concordance in monozygotic twins, which suggests a genetic contribution to its pathogenesis [4]. It is thought that a possible genetic cause of insulin resistance (IR) in PCOS could be, among others, a high expression of Src homology 2 (SH2) domain-containing adaptor protein (Lnk) in the ovarian cell lines of PCOS women, which inhibits phosphatidylinositol 3 kinase (PI3K) and mitogen-activated protein kinase (MAPK) signaling in response to insulin [5]. Intriguingly, Sam68, a member of the family of RNA-binding proteins, can interact with several proteins containing the SH2 and SH3 domains through the tyrosine-phosphorylated and proline-rich sequence residues, respectively [6]. Therefore, this protein is a scaffold protein recruited in various signal transduction pathways [7,8]. In particular, the tyrosine phosphorylation of Sam68 has been previously implicated in cell proliferation and growth, as well as in differentiation processes through different mechanisms. In fact, Sam68 phosphorylation has been reported to be stimulated by mitogenic and trophic hormones such as insulin and leptin in various biological systems [9], where it has been linked to cellular growth and differentiation processes. Recently, we have demonstrated the participation of Sam68 in leptin receptor signaling (via MAPK and PI3K) [10], mediating the leptin effect on aromatase expression in granulosa cells (GCs). More specifically, we showed a lower expression of Sam68 in granulosa cells from women with PCOS, which resulted in a lower activation of the MAPK and PI3K pathways that were resistant to the leptin effect on aromatase expression. As Sam68 has been related to both leptin and insulin-dependent PI3K and MAPK pathway activation in different cellular systems and there is not much information related to the expression of Sam68 in insulin signaling in GCs from healthy and PCOS women, we aim to investigate the role of this protein in the signal transduction pathways that are activated by insulin in the GCs from healthy donors. This is important as it is known that the insulin resistance, observed predominantly in PCOS women, may contribute to the ovulatory dysfunction [11]. Moreover, Sam68 plays a highly specialized role in the gonads, where its ablation causes infertility and female severe subfertility [12,13]. Therefore, IR could modulate the activity and/or expression levels of Sam68 and vice versa, which could probably mediate various pathological situations.

## 2. Materials and Methods

The study was approved by the local IRB (protocol 0008-N-16), and the women signed an informed consent. We included women with PCOS (n = 25) and healthy donors (n = 25), within the age range of 20 to 42 years, from GINEMED, Assisted Reproduction Centre, Seville, Spain. All the women were evaluated through a standardized screening protocol which has been described in detail elsewhere [14]. PCOS was diagnosed according to the Rotterdam criteria in the presence of two or more of the following criteria: oligo- and/or anovulation, clinical and/or biochemical signs of hyperandrogenism, and polycystic ovarian morphology, as assessed by transvaginal ultrasound [1]. Women with endometriosis and poor ovarian response were excluded from the study. The characteristics of the healthy donors and PCOS patients are shown in Table 1.

### 2.1. Human Granulosa Cell Isolation and Culture

Granulosa cells (GCs) from follicular aspirates were isolated using the protocol described in the literature by Ferrero et al. [15]. The GCs from healthy donors were seeded in six-well dishes and incubated overnight (37 °C, 5% CO_2_) to enable the removal of non-adherent cells. Subsequently, the GCs from each patient were washed and cultured for 24 h in McCoy’s medium (BioWhittaker^®^, Walkersville, MD, USA), supplemented with penicillin, 100 mg/mL streptomycin, and 10% fetal calf serum (FCS), 100 U/mL at 37 °C in 5% CO_2_. Next, the GCs were treated with or without insulin (0.1–10 nM) for 24 h (experiments of expression) or 10 min (experiments of inhibition) in a medium without FCS. Insulin was provided by Sigma (Sigma Chemical, St. Louis, MO, USA); a 10 nM dose of insulin was used for the experiments of the inhibition of Sam68, corresponding to our previously described results of optimal dose response [16].

The cell lysates were washed with cold PBS and solubilized for 30 min at 4 °C in lysis buffer containing 20 mM Tris, pH 8, 1% Nonidet P-40, 137 mM NaCl, 1 mM MgCl_2_, 1 mM CaCl_2_, 10% glycerol, 1mM phenylmethylsulfonyl fluoride, and 0.4 mM sodium orthovanadate. The total protein levels were determined by the bicinchoninic acid method [17], using bovine serum albumin as standard.

### 2.2. Immunoprecipitation

The soluble cellular lysates (0.5 mg of protein) from GCs were precleared with 50 μL of protein A-sepharose (Pharmacia, Uppsala, Sweden) for 2 h at 4 °C by end-over-end rotation.

The precleared cellular lysates were incubated with rabbit anti-Sam68 (C-20) 1:1000 from Santa Cruz Biotechnology (Dallas, TX, USA) for 3 h at 4 °C [18]. Next, 50 μL of protein A-sepharose was added to the immune complexes and incubation continued for 2 h. The immunoprecipitates were washed three times with lysis buffer. We added 30 μL of SDS-stop buffer containing 100 mM dithiothreitol (DTT) to the immunoprecipitates, followed by boiling for 5 min. The soluble supernatants were then resolved by Western blotting by using 7–10% SDS-PAGE and electrophoretically transferred onto nitrocellulose membranes [19].

Western blot analysis: The total cell lysates and immunoprecipitates were prepared in lysis buffer. The lysates were centrifuged at 10,000× *g* for 10 min to remove cellular debris. The protein concentration of the supernatant was determined by the Bradford staining method, with bovine serum albumin (BSA) as standard. The lysates were mixed with Laemmli’s sample buffer containing 2% SDS and 30 mM β-mercaptoethanol, boiled for 5 min, resolved by SDS-PAGE on a 12% gel, and electrophoretically transferred to a nitrocellulose membrane (Hybond, Amersham Pharmacia, Amersham, UK). The membranes were equilibrated in 1× PBS, and the non-specific binding sites were blocked by 5% non-fat milk in PBS at room temperature for 1 h. The membranes were then immunoblotted with antibody for 1 h (rabbit anti-Sam68 (C-20) 1:1000 from Santa Cruz Biotechnology; the anti phosphotyrosine (4G10) 1:1000 from Millipore (Burlington, MA, USA); mouse anti-phospho-ERK1/2 (pT202-Y204/pT185-Y187) 1:1500; mouse anti-phospho-AKT (pS473) 1:1000; and mouse anti-phospho-IRS-1 1:000 (pT1222) were from Cell Signaling Technology (Danvers, MA, USA). Loading controls were performed by immunoblotting the same membranes with polyclonal rabbit anti-GAPDH (1:2500, Calbiochem). The antibodies were detected using horseradish peroxidase-linked goat anti-rabbit/anti-mouse IgG (1:12,000, Amersham) and visualized using a highly sensitive chemiluminescence system (Supersignal, Pierce). Quantification of the protein bands was determined by densitometry using Image Gauge version 3.12 software (ScienceLab, Fuji Photo Film Co., Ltd., Tokyo, Japan).

### 2.3. Transfection Experiments

The GCs from the healthy donors were plated onto six-well dishes containing 2 mL of DMEN-F12 medium plus 10% FCS and incubated overnight (37 °C, 5% CO_2_) to enable the removal of non-adherent cells. The medium was replaced, and the transfection of cells was performed. For the experiments involving gene silencing, the cells were transfected with 2 μg of siRNA oligonucleotides of Sam68 (Integrated DNA technology, Inc., Coralville, IA, USA), using LipofectAMINE (Life Technologies, Carlsbad, CA, USA) transfection reagent according to the manufacturer’s instructions.

Duplex Sequences: Forward, 5′-CGCAGAACAAAGUUACGAAGGCUAC-3′; reverse, 5′-GUAGCCUUCGUAACUUUGUUCUGCGUA-3′.

For the experiments involving overexpression of Sam68, we used a pcDNA3 vector expressing Sam68 plasmid; between 1 and 3 ug of DNA was transfected into the GCs from the women with PCOS.

Following transfection, the medium was replaced with a serum-free medium for another 24 h, and the cells were stimulated with or without 10 nM insulin for 10 min. Transfection analyses were performed by duplicate in each of at least three independent experiments.

### 2.4. RNA Extraction and Quantitative Real-Time PCR (qRT-PCR) Assay

The relative abundance of Sam68 mRNA (gene *Khdrbs1*), insulin receptor (gene *INSR*), and IRS-1 (gene *IRS-1*) was determined by qRT-PCR, as previously described [20]. qRT-PCR was performed using the following primers based on the sequences of the National Center for Biotechnology Information GenBank database:

Sam68 (GeneBank accession: NM_006559.3): forward, 5′-TTTGTGGGGAAGAT TCTTGG-3′; reverse, 5′ GGGGGTCCAAAGACTTCAAT-3′.

Cyclophilin (GeneBank accession: NM_000942): forward, 5′ CTTCCCCGATACTTCA-3′; reverse, 5′-TCTTGGTGCTACCTC-3′3′.

IRS-1 (GeneBank accession: JX901289.1): forward, 5′-ATGGCGAGAGCCCTCCGGATACC-3′; reverse, 5′-CTCATAATACTCCAGGCGCGC-3′.

INSR (GeneBank accession: AH002851.2): forward, 5′TTCACTGGCAATCGCATTGAGCTG-3′; reverse, 5′-TCATGGGTCACAGGGCCAATGATA-3′.

The Opticon Monitor 3 Program was used to determine the threshold cycle (CT) from each well. Relative quantification was calculated using the 2^−∆∆CT^ method. For the treated samples, evaluation of 2^−∆∆CT^ indicates the fold change in gene expression, normalized to housekeeping genes (Cyclophilin), and relative to the untreated cells (0% FBS). The specificity of the amplifications was confirmed by melting curves analysis. Reaction mixtures without reverse transcriptase or RNA were run in parallel to ensure the absence of sample contamination.

### 2.5. Data Analysis

The experiments were repeated separately at least three times to assure reproducible results. The results are expressed as the mean ± standard deviation (S.D.). The statistical significance was assessed by Student’s test or, when necessary, by one-way ANOVA, followed by Bonferroni’s multiple comparison post-hoc test. Differences between groups were considered significant at *p*-value < 0.05, using the Graph Pad Instat computer program (San Diego, CA, USA).

## 3. Results

### 3.1. Insulin Increased Sam68 Phosphorylation and mRNA Expression in Granulosa Cells from Healthy Donors

**Figure 1:**
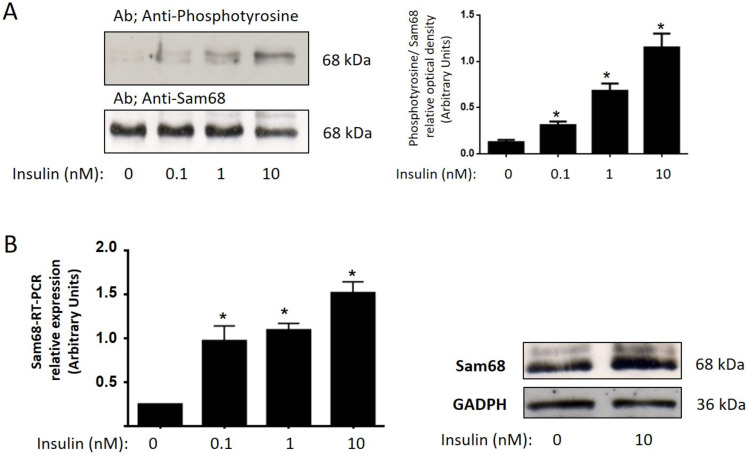
Insulin increases Sam68 phosphorylation in human GCs from healthy donors. (**A**) GCs were incubated in absence or presence of insulin (0.1–10 nM) for 10 min, lysed, and the soluble clarified cell lysates were subjected to immunoprecipitation with anti-Sam68 antibodies. Immunoprecipitates were resolved by SDS-PAGE and Western blot with anti-phosphotyrosine antibodies. The lysates were analyzed by immunoblot using the anti-Sam68 antibodies to control the amount of protein in every lane. Statistical analyses were performed by one-way ANOVA followed by Bonferroni’s multiple comparison post-hoc test. A representative experiment run in duplicates from 3 different donors is shown. Densitograms with SD are shown; * *p* < 0.05 versus control. (**B**) Total RNA was extracted as described in Section 2, and Sam68 mRNA was quantified with qRT-PCR in independent experiments. Cyclophilin was used as internal standard. Results shown are expressed as means ± SD from three independent experiments, run in triplicates; * *p* < 0.05 versus control. Statistical analyses were performed by one-way ANOVA. Asterisks indicate significant differences from the control according to Bonferroni’s multiple comparison post-hoc test.

We previously demonstrated the Tyr-phosphorylation of Sam68 in response to insulin in different cell systems [21]. Now, to assess whether Sam68 is involved in insulin’s action on human GCs from healthy donors, we incubated GCs for 10 min in a medium with or without different insulin concentrations (0.1–10 nM) (Figure 1). Insulin (Figure 1A) increased the Sam68 phosphorylation in a dose-dependent manner, in GCs, as determined by Western blot analysis. This effect was dose-dependent, and the maximal effect was achieved at 10 nM insulin. The amount of total protein in every sample was controlled using anti-Sam68 antibodies.

In order to further study the effects of insulin on Sam68 expression, qRT-PCR analysis was carried out using cyclophilin as an internal control. The GCs were independently incubated in the absence of serum with and without insulin (0.1–10 nM) for 24 h. As shown in Figure 1B, the expression level of the Sam68 gene was increased in response to insulin, dose-dependently, and the maximal effect was achieved at 10 nM insulin.

The Sam68 expression in response to insulin was confirmed by immunoblot of the cell lysates of the control granulosa cells using anti-Sam68 antibodies (Figure 1).

### 3.2. Sam68 Downregulation Prevents Insulin Activation of Signaling Pathways in Granulosa Cells from Healthy Donors

Insulin receptor is known to activate phosphatidylinositol-3 kinase (PI3K) and mitogen-activated protein kinase (MAPK). To test the effects of Sam68 downregulation on insulin signaling, human GCs from healthy donors were used and immunoblotting was employed to analyze the phosphorylation of the kinases.

**Figure 2:**
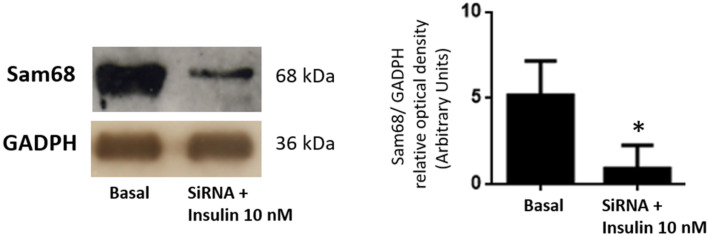
Control of inhibition of Sam68 by using siRNA in human GCs from healthy donors. GCs were transfected with Sam68 siRNA or NC1-scrambled negative control siRNA (basal) duplexes, as described in Section 2, and incubated in absence or presence of 10 nM insulin for 10 min. GCs lysates were separated by SDS-PAGE and Western blot analysis was performed by using anti-Sam68 antibodies to study sam68 expression. Sample protein loading was controlled by using anti-GAPDH antibodies. We show the corresponding densitometric analysis of three independent experiments as means ± SD; statistical analyses were performed by ANOVA. Asterisks indicate significant differences from the un-treated controls according to Bonferroni’s multiple comparison post-hoc test.

The cells were grown to 60–70% confluence in 6-well dishes and were first transfected using Sam68 siRNA or NC1 negative control siRNA duplexes and incubated in the absence or presence of insulin for 10 min, as previously indicated in Material and Methods. Both anti-Sam68 and anti-GAPDH antibodies were used as a control of Sam68 downregulation and loading control, respectively. This approach achieved a decrease in the Sam68 expression ranging from 60–70% of the control value (Figure 2).

**Figure 3:**
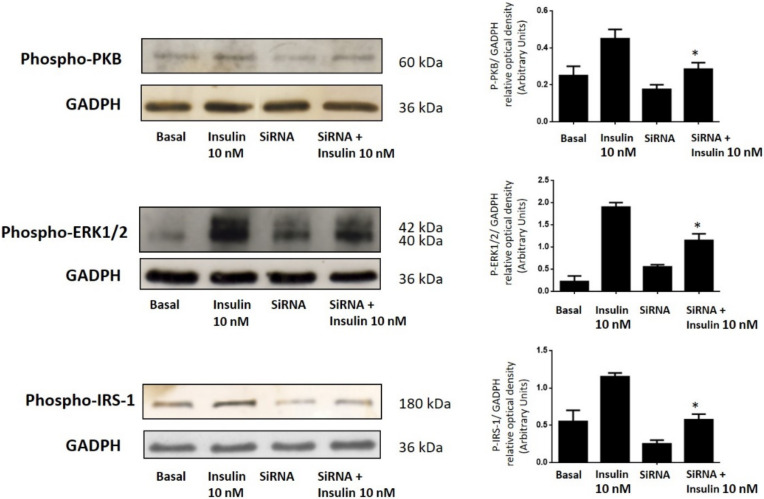
Sam68 siRNA prevents the insulin-dependent activation of PI3K and MAPK pathways in human GCs from healthy donors. GCs were transfected with Sam68 siRNA or NC1-scrambled negative control siRNA (basal) duplexes, as described in Section 2, and incubated in absence or presence of 10 nM insulin for 10 min. GC lysates were separated by SDS-PAGE, and Western blot analysis was performed by using anti-P-PKB, anti-P-ERK1/2, and anti-P-IRS-1 antibodies to study insulin activation of the PI3K and MAPK signaling pathway. Sample protein loading was controlled by using anti-GAPDH antibodies. We show the corresponding densitometric analysis of three independent experiments as means ± SD; statistical analyses were performed by ANOVA. Asterisks indicate significant differences from the untreated controls according to Bonferroni’s multiple comparison post-hoc test.

We measured the activation of the PI3K pathways by employing antibodies that specifically recognize the phosphorylated forms of the central kinase PKB. Moreover, we also measured the activation of the MAPK pathways by employing antibodies that specifically recognize the phosphorylated forms of ERK1/2. As shown in Figure 3, insulin-mediated PKB and ERK1/2 phosphorylation was significantly reduced in the GCs where the Sam68 was downregulated. This effect was almost completely abolished by decreasing the expression of Sam68, suggesting the role of Sam68 in the insulin signaling pathways in the GCs.

Finally, to connect Sam68 expression with the mechanistic effect that it exerts over the main pathways of PI3K and MAPK under insulin stimulation, we next focus on the effect of siRNA Sam68 downregulation on the phosphorylated forms of insulin receptor substrate-1 (IRS-1) in human GCs. As was demonstrated using immunoblotting analysis with anti-phospho-IRS-1 antibodies, the downregulation of Sam68 in GCs significantly decreased the phosphorylation of IRS-1 in response to insulin (Figure 3).

### 3.3. Decreased Sam68, as Well as IR and IRS-1 Expression in Human GCs from PCOS Women Compared to GCs from Healthy Donors

**Figure 4:**
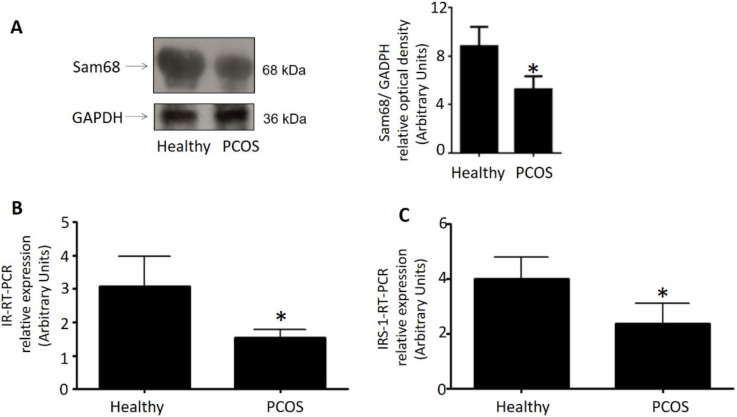
Diminished expression of Sam68, IR, and IRS-1 in GCs from PCOS women. GCs samples were obtained from 25 healthy donors and 25 PCOS women. (**A**) Representative Western blot analysis of Sam68 protein level in GCs from healthy donors and PCOS women. GC lysates were denatured and resolved by SDS-PAGE with anti-Sam68 antibodies, as described in Section 2. Loading controls were performed in the same membranes with anti-GAPDH. We show the corresponding densitometric analysis of three independent experiments as means ± SD. Statistical analyses were performed by ANOVA. (**B**) Relative mRNA level of IR in GCs from healthy donors and PCOS women, (**C**) Relative mRNA level of IRS-1 in GCs from healthy donors and PCOS women. Both IR and IRS-1 mRNA were quantified with qRT-PCR. RNA was extracted as described in Section 2. Cyclophylin was used as internal standard. Results shown are expressed as means ± SD from three independent experiments, run in triplicates; * *p* < 0.05 versus control. Statistical analyses were performed by ANOVA. Asterisks indicate significant differences from the control according to Bonferroni’s multiple comparison post-hoc test.

As Sam68 protein is highly expressed in the gonads, we also aimed to compare the expression of Sam68 in the GCs from patients diagnosed with PCOS versus those obtained from healthy donors, used as controls. We evaluated the Sam68 expression by means of Western blot analysis, using GAPDH as a control. As shown Figure 4A, the Sam68 expression was lower in the GCs from PCOS women than in the GCs from the healthy donors, suggesting a role played by Sam68 in these cells, which might allow this protein to affect different biological processes, such as the insulin signaling pathway. That is why we also aimed to compare the expression of IR in the GCs from PCOS versus healthy donors. In this case, IR mRNA was quantified with qRT-PCR, using Cyclophylin as an internal standard. As shown Figure 4B, the IR expression was lower in GCs from PCOS women than in the GCs for healthy donors, suggesting a role played by Sam68 in these cells. Moreover, in order to further study the effect of Sam68 on IR expression, we also compared the expression of IRS-1 in the GCs from PCOS versus healthy donors by means of qRT-PCR and using Cyclophylin as an internal standard. As shown in Figure 4C, the IRS-1 expression was lower in the GCs from the PCOS women than in the GCs from the healthy donors, suggesting a role played by Sam68 in the insulin signaling pathways in granulosa cells.

### 3.4. Sam68 Overexpression Increases Inulin Activation of Signaling Pathways in Human GCs from PCOS Women

**Figure 5:**
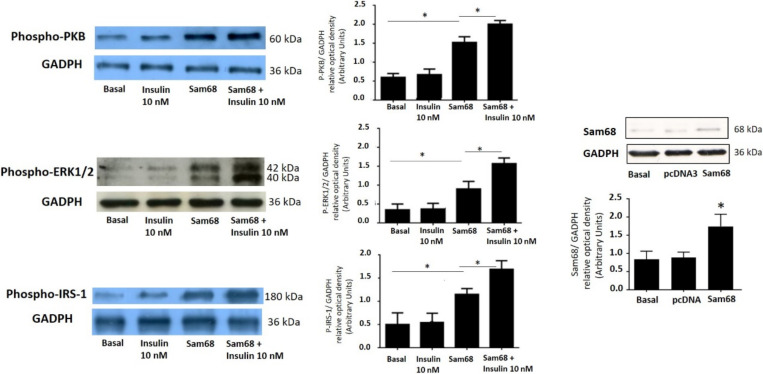
Sam68 overexpression increases the insulin-dependent activation of PI3K and MAPK path-ways in human GCs from women with PCOS. GCs were transfected with Sam68 plasmid, as described in Section 2, and incubated in absence or presence of insulin 10 nM for 10 min. GC lysates were separated by SDS-PAGE, and Western blot analysis was performed by using anti-P-IRS-1, anti-P-PKB, and anti-P-ERK1/2 antibodies to study insulin activation of the PI3K and MAPK signaling pathways. Sample protein loading was controlled by using anti-GAPDH antibodies. Control of transfection of Sam68 is shown by using pcDNA3 plasmid with or without Sam68 cDNA as control in human GCs from PCOS. We show the corresponding densitometric analysis of three independent experiments as means ± SD. Statistical analyses were performed by ANOVA. Asterisks indicate significant differences from the control according to Bonferroni’s multiple comparison post-hoc test Sam68 expression was confirmed by immunoblot of cell lysates of granulosa cells in the absence and presence of 10 nM insulin for 24 h, using anti-Sam68 antibodies and anti-GAPDH as the loading control.

Finally, to further study the effect of Sam68 in insulin signaling pathways, we investigated the upregulation of Sam68 by transfecting the GCs from PCOS women with a pcDNA3 vector expressing the Sam68 plasmid. Following transfection for 24 h, the medium was replaced with serum-free medium for another 24 h. Next, the GCs were incubated in the absence or presence of 10 nM insulin for 10 min. As shown in Figure 5, the upregulation of Sam68 increased the insulin-dependent activation of the PI3K and MAPK pathways in the GCs from the PCOS women. More specifically, the upregulation of Sam68 increased the phosphorylation of IRS-1, PKB, and ERK1/2, suggesting the role of Sam68 in promoting greater insulin sensitivity in GCs. The overexpression of Sam68 was confirmed by immunoblot (Figure 5).

## 4. Discussion

PCOS is one of the most common reproductive and metabolic disorders among women of reproductive age. Women suffering from PCOS present with a constellation of symptoms associated with hyperandrogenism, which significantly impacts their quality of life. They may be at increased risk of multiple morbidities, including obesity and insulin resistance. In fact, defects in insulin actions or in the insulin signaling pathways are central in the pathogenesis of the syndrome. Most women with PCOS are metabolically insulin-resistant, in part due to genetic predisposition and in part to environmental factors such as obesity. Therefore, a pivotal role in the pathophysiology of this syndrome is played both by aberrant insulin signaling as well as by visceral adiposity [22,23]. Indeed, decreased insulin sensitivity, attributable to a post-receptor binding defect in the insulin signaling pathways, has been identified as an intrinsic component of PCOS, independently of obesity [24]. An alteration in the gene expression of some players in the insulin signaling pathways by microarray gene analysis has also been reported [25,26]. However, despite the progress achieved in the diagnosis and management of PCOS, not much is known about the molecular players and the signaling pathways underlying its mechanisms, and the primary origin still remains an unsolved issue. That is why constant efforts are being made to understand this complex pathogenetic network, including the developmental origins of the syndrome, obesity, and insulin resistance, as well as its association with reproductive and metabolic disturbances. In order to clarify this complex pathogenesis, we aim to study the expression of Sam68 in response to insulin in the GCs from healthy donors. This is a protein whose function in reproduction and fertility has been suggested [27,28]; moreover, Sam68 could be recruited in various signal transduction pathways [8,29], including dependent PI3K and MAPK activation insulin and leptin signaling [30,31]. In this sense, recently, we have demonstrated a decrease in the aromatase gene expression in response to leptin in the GCs from PCOS donors compared to healthy donors, possibly due to the lower expression of Sam 68 found in the GCs of donors with PCOS. Therefore, the lower activity of aromatase (necessary for the formation of estrogen) activity reported in women with PCOS could be the reason for the elevated level of androgen (the most common endocrine disorder associated with PCOS). As Sam68 is a protein, whose structural characteristics allow multiple types of post-translational modifications, we aimed to study whether acute insulin administration affects the tyrosine phosphorylation of Sam68 in GCs from healthy donors, as previously shown in other cell types [32]. This effect of insulin is dose-dependent, in a similar way to that previously observed with leptin in the same system. As a result of this tyrosine phosphorylation of Sam68 in response to insulin, the RNA binding capacity of Sam68 could be diminished in GCs, which would be consistent with previously reported data demonstrating that the tyrosine phosphorylation of Sam68 by kinases of the Src [33] and Brk [34,35] family negatively regulates its RNA binding function. However, the mechanism whereby the tyrosine phosphorylation of Sam68 negatively regulates its RNA binding function is intriguing and remains to be investigated in GCs.

Next, the possible participation of Sam68 in insulin receptor signaling and the regulation of Sam68 expression by insulin in GCs from healthy donors were also investigated. In this context, we also found that the insulin stimulation of GCs increased the expression of Sam68 in a similar way to that previously observed with leptin in GCs from healthy donors [36] and other systems [9]. Therefore, this result further supports the possible role of Sam68 in the signaling of insulin in GCs. Because, as previously shown in many different cellular systems, insulin activates both MAPK and PI3K pathways, by now using the silencing gene expression strategy, we have also demonstrated that Sam68 is mediating the insulin action in GCs by the participation in the insulin-dependent activation of the MAPK and PI3K signaling pathways. Regarding the mechanism whereby Sam68 may mediate the activation of these pathways, it has previously been reported that Sam68 is associated with the SH2 and SH3 domains of proteins [33], suggesting a role of Sam68 in the MAPK pathway. Moreover, the association of Tyr-phosphorylated Sam68 with the regulatory subunit of PI3K has been previously demonstrated in GCs in response to leptin [36]. This interaction may enhance the activation of the PI3K pathway, which may also support a role of Sam68 in the activation of this pathway by insulin. However, this interaction remains to be confirmed in coprecipitation studies. Additionally, IRS-1 is a key protein linking the PI3K and MAPK signaling pathways [37]. Therefore, as Sam68 seems to regulate IRS-1 expression, as we observed in the Sam68 downregulation experiments, the role of Sam68 stimulating the PI3K and MAPK signaling pathways may also be mediated by IRS-1 in GCs. However, the mechanism whereby Sam68 may modulate IRS-1 expression is intriguing and also remains to be investigated. Thus, Sam68 plays a role in the transduction of the insulin signal from the plasma membrane to the RNA metabolism via a rapid mechanism mediated by phosphorylation, and therefore, some of the effects of insulin in GCs may be mediated, at least in part, by modulation of the RNA metabolism.

Insulin is a well-characterized factor with biological effects on survival, growth, and proliferation in different biological systems [38,39,40]. Insulin resistance is prevalent in women with PCOS independently of obesity and is critically involved in reproductive and metabolic complications of the syndrome. However, some studies have shown contradictory results regarding the role of insulin in GCs from PCOS donors [41]. Thus, the investigation of the insulin action in GCs from women with or without PCOS is relevant in further understanding this problem. In this context, we found that Sam68 is downregulated in GCs from PCOS women, and as a consequence, the resistance to insulin action in GCs from PCOS women may be partly mediated by the lower expression of Sam68. Moreover, the overexpression of Sam68 in granulosa from PCOS women increased insulin signaling. Similar findings were also observed with leptin in GCs from PCOS [36]. Intriguingly, insulin stimulates leptin gene expression, enhancing leptin secretion [42]. Taken all together, it could be speculated that the lower expression of Sam68 in PCOS may, in part, be responsible for insulin resistance, and as a consequence, the resistance to leptin action on aromatase expression in GCs from PCOS women [36]. This is important as hyperandrogenism is a critical factor of the pathophysiologic changes and clinical features associated with PCOS [42]. Therefore, the current study further supports the role of Sam68 in PCOS and provides, for the first time, a novel mechanistic insight into the mechanism underlying the insulin resistance in PCOS.

In conclusion, PCOS might represent a common end-stage clinical phenotype of different processes, in which there is impaired insulin action, probably favored by specific, intrinsic abnormalities in these women. Our results suggest the participation of Sam68 in insulin receptor signaling, mediating, possibly, the insulin effect in GCs and pointing to a new target in insulin resistance observed in GCs from PCOS women.

## Figures and Tables

**Table 1:** Characteristics of the healthy donors and PCOS patients.

	Healthy	PCOS
**Patients (n)**	25	25
**Female age, yrs.**	27	32
**BMI (kg/m^2^) (%)**	22	24.3
**Smokers**	No	No
**LH/ FSH ratio**	<2.0	>3.0
**Endometriosis (%)**	No	No

## Data Availability

Anonimous data are available upon reasonable request.
